# Downregulation of JKAP is correlated with elevated disease risk, advanced disease severity, higher inflammation, and poor survival in sepsis

**DOI:** 10.1002/jcla.22945

**Published:** 2019-06-17

**Authors:** Min Zhao, Xing Huang

**Affiliations:** ^1^ Department of Emergency Medicine The Central Hospital of Wuhan, Tongji Medical College, Huazhong University of Science & Technology Wuhan China

**Keywords:** disease risk, inflammation, JNK pathway–associated phosphatase, sepsis, survival

## Abstract

**Objective:**

This study aimed to explore the association of JKAP with sepsis risk and investigate its correlation with disease severity, inflammatory cytokines, and survival in sepsis patients.

**Methods:**

A hundred and one sepsis patients along with 100 healthy controls were enrolled, and their blood serum samples were collected for JKAP and inflammatory cytokines measurement by enzyme‐linked immunoassay. The difference in serum JKAP between sepsis patients and healthy controls was determined. Among sepsis patients, the correlation of JKAP with disease severity, laboratory indexes, inflammatory cytokines, 28‐day mortality, and accumulating survival was analyzed.

**Results:**

JNK pathway–associated phosphatase level was decreased in sepsis patients compared with healthy controls and presented with good value in predicting decreased sepsis risk (AUC = 0.896 [95% CI: 0.851‐0.941]). And its low expression was associated with advanced disease severity (APACHE II score and SOFA score) and systemic inflammation (CRP, PCT, TNF‐α, IL‐1β, IL‐6, and IL‐17) in sepsis patients. Additionally, JKAP level was decreased in deaths compared with survivors and had good value in distinguishing deaths from survivors (AUC = 0.742 [95% CI: 0.636‐0.849]). Further, Kaplan‐Meier curve analysis disclosed that JKAP high expression predicted more prolonged accumulating survival in sepsis patients.

**Conclusion:**

JNK pathway–associated phosphatase is of good value in predicting lower sepsis risk, and its downregulation correlates with advanced disease severity, higher level of systemic inflammation, and poor survival in sepsis patients.

## 1 INTRODUCTION

Sepsis is defined as a life‐threatening systemic inflammatory response syndrome caused by dysregulated host responses to infection.^1^, ^2^ As a leading cause of death among critically ill patients, sepsis has an estimated 1% rise in incidence per year and affects over 18 million people worldwide .^3^ Meanwhile, in China, the hospital and intensive care unit (ICU) mortality in severe sepsis ranges from 33.5% to 48.7%, which can be explained by the lack of effective early diagnosis, treatment delay, and high prevalence of nosocomial infection in the ICU.^3^, ^4^ Effective treatments in sepsis rely on timely removal of the source of infection and appropriate choice of antimicrobial therapy, whereas there are still a large number of sepsis patients die from delayed diagnosis and ineffective treatment. Therefore, the exploration of potential biomarkers, which can promote sensitivity and specificity of diagnosis and prognosis, is essential to achieve optimized treatment outcomes and increased survival in sepsis patients.^5^, ^6^

JNK pathway–associated phosphatase (JKAP), also known as dual‐specificity phosphatase (DUSP) 22, serves as a tyrosine phosphatase and specifically activates kinases JNK.^7^ It is expressed in various types of human cells such as T cells, B cells, and natural killer cells, suggesting that JKAP is possibly involved in immune and inflammation response of human diseases.^8^, ^9^ Moreover, evidence reveals that JKAP is downregulated and negatively correlates with inflammation level and disease activity in systemic lupus erythematosus (SLE) as well as inflammatory bowel disease (IBD).^8^, ^9^ Considering the above‐mentioned data which indicate that JKAP is a key factor in regulating immune, inflammation and is closely involved in the etiology of several inflammatory diseases, we hypothesize it might participate in the development and progression of sepsis. However, no related research has been reported until now. Thus, we performed this study to explore the association of JKAP expression with sepsis risk and investigate its correlation with disease severity, inflammatory cytokines, and survival in sepsis patients.

## 2 MATERIALS AND METHODS

### 2.1 Participants

This study consecutively recruited 101 sepsis patients who were admitted to the Department of Emergency Medicine of The Central Hospital of Wuhan between January 2016 and February 2018. The inclusion criteria were as follows: (a) diagnosed as sepsis according to the Surviving Sepsis Campaign: International Guidelines for Management of Severe Sepsis and Septic Shock, 2012^10^; (b) age above 18. The exclusion criteria were as follows: (a) severe primary disease including malignancies and human immunodeficiency virus (HIV); (b) died within 24 hours after admission to the Department of Emergency Medicine; (c) receiving immunosuppressant before enrollment; and (d) pregnant or lactating women. In addition, 100 healthy individuals who had no history of severe infection or malignancies, or other obvious abnormalities by physical examination were enrolled as controls. The study protocol was approved by the Ethics Committee of The Central Hospital of Wuhan. All patients or their guardians and healthy controls signed informed consents.

### 2.2 Data collection

After the enrollment, all patients’ data were collected, which consisted of the following: (a) demographic data: age, gender, BMI, and smoke; (b) chronic comorbidities: chronic obstructive pulmonary disease (COPD), cardiomyopathy, chronic kidney failure, and cirrhosis; (c) laboratory indexes: serum creatinine (Scr), albumin, white blood cell (WBC), C‐reactive protein (CRP), and procalcitonin (PCT).

### 2.3 Assessments and follow‐up

Acute physiology and chronic health evaluation (APACHE) II score and sequential organ failure assessment (SOFA) score were assessed within 24 hours after admission to the Department of Emergency Medicine. For the APACHE II score, the evaluation was composed of three parts, including physiologic scores ranging from 0 to 60 points, age scores ranging from 0 to 6 points, and comorbid disease scores ranging from 0 to 5 points, with a distribution of 0 to 71 points. The severity was increased when the APACHE II score was higher.^11^ The SOFA scoring scheme assigned 1 to 4 points to each level of dysfunction of six organs (respiratory, circulatory, renal, hematological, hepatic, and central nervous systems). A score of 0 to 24 points was assigned, and a higher score was associated with an increased severity.^12^ After enrollment, all patients were monitored daily and consecutively followed up for 28 days, and the 28‐day mortality was recorded. Accumulating survival was calculated from the day of admission to the day of death in the hospital or last visit.

### 2.4 Samples collection

Blood samples of sepsis patients were collected within 24 hours after admission, and those of healthy controls were collected on the enrollment. For separation of serum, the centrifugation was performed at the condition of 3000 r/min，15 minutes，4°C, within 1 hour after the blood samples collection. After the centrifugation, the serums were stored at −80°C until determination.

### 2.5 Detection of JKAP and inflammatory cytokines in serum

The JKAP levels in serum of sepsis patients and healthy controls were determined by enzyme‐linked immunoassay (ELISA) with human ELISA Kits designed by Shanghai Enzyme‐linked Biotechnology Co., Ltd. (Enzyme‐linked Biotechnology). The inflammatory cytokines expressions including tumor necrosis factor (TNF‐α), interleukin‐1β (lL‐1β), IL‐6, and IL‐17 in the serum of sepsis patients were determined by ELISA with commercial human ELISA Kits (eBioscience). All the procedures of ELISA were performed in accordance with the manufacturer's instructions.

### 2.6 Statistical analysis

Data of this study were presented as mean (standard deviation [SD]), count (%), or median (interquartile range [IQR]); Wilcoxon rank‐sum test was performed to examine the differences between groups; and Spearman's rank correlation test was used to perform the correlation analysis. Receiver operating characteristic (ROC) curves were generated by plotting the sensitivity against one‐specificity, and the area under the curve (AUC) with 95% confidence interval (CI) was calculated to assess the ability of JKAP level to discriminate between sepsis and healthy controls, or the value of JKAP level in predicting 28‐day mortality of sepsis. Kaplan‐Meier curve was used to illustrate accumulating survival, and the survival difference between two groups was determined by log‐rank test. Univariate and multivariate logistic regression model analyses were performed to determine predicting factors of 28‐day mortality. Univariate and multivariate Cox's proportional hazards regression model analyses were used to screen for affecting factors of accumulating survival during the study. Statistical analysis was performed using SPSS 22.0 (SPSS Inc), and figures were plotted using GraphPad Prism 7.00 (GraphPad Software). *P* value < 0.05 was considered as significant.

## 3 RESULTS

### 3.1 Clinical characteristics

Among all the sepsis patients (N = 101), the mean age was 55.3 ± 12.2 years and the number of males and females was 70 and 31, respectively (Table 1). As for disease severity, the mean APACHE II score was 13.97 ± 6.22 and the mean SOFA score was 5.84 ± 3.10. Regarding laboratory indexes, the median levels of Scr, Albumin, WBC, CRP, and PCT were 1.68 (1.14‐2.30) mg/dL, 25.25 (21.27‐35.33) g/L, 17.20 (3.04‐28.31) 10^9^/L, 105.33 (54.15‐153.65) mg/L, and 15.10 (8.51‐28.26) ng/mL, respectively. With respect to inflammatory cytokines levels, the median values of TNF‐α, IL‐1β, IL‐6, and IL‐17 were 213.35 (129.63‐327.22) pg/mL, 13.59 (5.75‐28.64) pg/mL, 62.63 (40.95‐147.91) pg/mL, and 150.04 (65.50‐224.37) pg/mL, respectively. In addition, the median of JKAP level was 12.89 (6.49‐20.27) pg/mL. Other detailed baseline characteristics of sepsis patients were shown in Table 1.

**Table 1:** Baseline characteristics of sepsis patients

Characteristics	Sepsis patients (N = 101)
Demographic characteristics	
Age (y, mean [SD])	55.3 (12.2)
Male sex, No. (%)	70 (69.3)
BMI, kg/m^2^, mean (SD)	22.6 (5.0)
Smoke, No. (%)	30 (29.7)
Chronic comorbidities, No. (%)	
COPD	16 (15.8)
Cardiomyopathy	35 (34.7)
Chronic kidney failure	9 (8.9)
Cirrhosis	19 (18.8)
Disease severity, mean (SD)	
APACHE II score	13.97 (6.22)
SOFA score	5.84 (3.10)
Laboratory indexes, median (IQR)	
Scr, mg/dL	1.68 (1.14‐2.30)
Albumin, g/L	25.25 (21.27‐35.33)
WBC, 10^9^/L	17.20 (3.04‐28.31)
CRP, mg/L	105.33 (54.15‐153.65)
PCT, ng/mL	15.10 (8.51‐28.26)
Inflammatory cytokines, median (IQR)	
TNF‐α, pg/mL	213.35 (129.63‐327.22)
IL‐1β, pg/mL	13.59 (5.75‐28.64)
IL‐6, pg/mL	62.63 (40.95‐147.91)
IL‐17, pg/mL	150.04 (65.50‐224.37)
JKAP level, pg/mL, median (IQR)	12.89 (6.49‐20.27)

Abbreviations: APACHE, acute physiology and chronic health evaluation; BMI, body mass index; COPD, chronic obstructive pulmonary disease; CRP, C‐reactive protein; IL, interleukin; IQR, interquartile range; JKAP, JNK pathway–associated phosphatase; PCT, procalcitonin; Scr, serum creatinine; SD, standard deviation; SOFA, sequential organ failure assessment; TNF, tumor necrosis factor; WBC, white blood cell.

### 3.2 Comparison of JKAP level between sepsis patients and healthy controls

**Figure 1:**
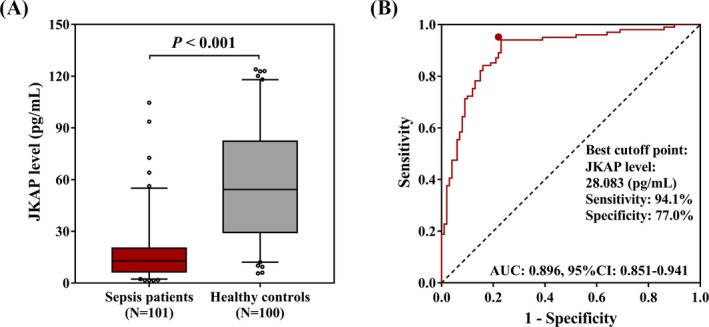
The predictive value of JKAP level in sepsis risk. The JKAP was decreased in sepsis patients compared with healthy controls (A). The comparison of JKAP level between sepsis patients and healthy controls was determined by Wilcoxon rank‐sum test. And JKAP was of great value in distinguishing sepsis patients from healthy controls (B). ROC curve was performed to evaluate the ability of JKAP level to discriminate sepsis patients from healthy controls. *P* < 0.05 was considered significant. JKAP, JNK pathway–associated phosphatase; ROC, receiver operating characteristic

The median of JKAP level in sepsis patients (N = 101) was 12.890 (6.485‐20.265) pg/mL, which was decreased compared with the healthy controls (N = 100) (54.228 [29.339‐82.223] pg/mL) (*P* < 0.001; Figure 1A). And ROC curve analysis exhibited that JKAP level was of great value in distinguishing sepsis patients from healthy controls with AUC of 0.896 (95% CI: 0.851‐0.941; Figure 1B). The sensitivity and specificity at the best cutoff point (the point where the sum of sensitivity and specificity was the largest) were 94.1% and 77.0%, respectively, and the cutoff value of JKAP was 28.083 pg/mL. These data indicated JKAP presented with potential to be a biomarker for decreased sepsis risk.

### 3.3 Correlation of JKAP level with disease severity, laboratory indexes, and inflammatory cytokines

In sepsis patients, JKAP level was negatively associated with both APACHE II score (*r* = −0.356, *P* < 0.001) and SOFA score (*r* = −0.343, *P* < 0.001; Table 2). With respect to laboratory indexes, JKAP level was negatively associated with Scr (*r* = −0.330, *P* = 0.001), CRP (*r* = −0.363, *P* < 0.001), and PCT (*r* = −0.267, *P* = 0.007). With regard to inflammatory cytokines, JKAP level was negatively associated with TNF‐α (*r* = −0.275, *P* = 0.005), IL‐1β (*r* = −0.279, *P* = 0.005), IL‐6 (*r* = −0.278, *P* = 0.004), and IL‐17 (*r* = −0.306, *P* = 0.002). These data indicated that JKAP negatively correlated with disease severity and systemic inflammation.

**Table 2:** Correlation of JKAP level with disease severity, laboratory indexes, and inflammatory cytokines

Items	JKAP level	
	*P* value	Correlation coefficient (*r*)
Disease severity		
APACHE II score	<0.001	−0.356
SOFA score	<0.001	−0.343
Laboratory indexes		
Scr	0.001	−0.330
Albumin	0.754	0.032
WBC	0.961	−0.005
CRP	<0.001	−0.363
PCT	0.007	−0.267
Inflammatory cytokines		
TNF‐α	0.005	−0.275
IL‐1β	0.005	−0.279
IL‐6	0.004	−0.278
IL‐17	0.002	−0.306

Note

Correlation analysis was determined by Spearman's rank correlation test. *P* value < 0.05 was considered significant.

Abbreviations: APACHE, acute physiology and chronic health evaluation; CRP, C‐reactive protein; JKAP, JNK pathway–associated phosphatase; PCT, procalcitonin; Scr, serum creatinine; SOFA, sequential organ failure assessment; TNF, tumor necrosis factor; IL, interleukin; WBC, white blood cell.

### 3.4 Comparison of JKAP level between deaths and survivors

**Figure 2:**
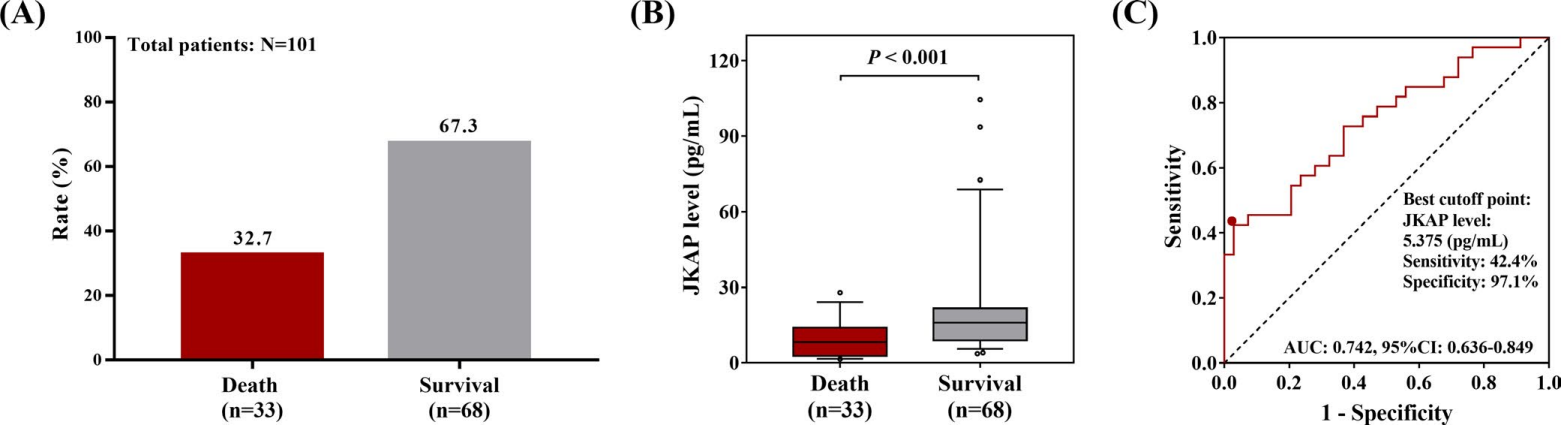
The prognostic value of JKAP level in sepsis. Death and survival accounted for 32.7% and 67.3% of total sepsis patients (A). Among all the sepsis patients, the JKAP level was decreased in death compared with survival (B). The comparison of JKAP level between death and survival was conducted by Wilcoxon rank‐sum test. JKAP was of value in determining death from survival in sepsis patients (C). ROC curve was conducted to assess the ability of JKAP to differentiate death from survival. *P* < 0.05 was considered significant. JKAP, JNK pathway–associated phosphatase; ROC, receiver operating characteristic

Among the 101 sepsis patients, the 28‐day mortality was 32.7% and the survival rate was 67.3% (Figure 2A). And the median JKAP level in deaths (8.150 [2.765‐13.950] pg/mL) was reduced compared with the survivors (15.900 [8.945‐21.645] pg/mL) (*P* < 0.001; Figure 2B). In addition, the ROC curve displayed that JKAP level was of good value in distinguishing the deaths from the survivors in sepsis patients with AUC of 0.742 (95% CI: 0.636‐0.849) with a sensitivity of 42.4% and a specificity of 97.1% at the best cutoff point, and the cutoff value of JKAP was 5.375 pg/mL (Figure 2C). These data indicated JKAP exhibited the potential to be a biomarker for decreased mortality.

### 3.5 Factors affecting 28‐day mortality by logistic regression model analyses

Univariate logistic regression revealed that JKAP level was negatively correlated with 28‐day mortality (OR = 0.232, *P* = 0.002), while APACHE II score (OR = 2.533, *P* = 0.036), SOFA score (OR = 7.407, *P* < 0.001), Scr (OR = 2.657, *P* = 0.026), WBC (OR = 2.353, *P* = 0.050), CRP (OR = 3.286, *P* = 0.008), PCT (OR = 2.857, *P* = 0.018), TNF‐α (OR = 3.810, *P* = 0.004), IL‐1β (OR = 6.810, *P* < 0.001), and IL‐17 (OR = 2.500, *P* = 0.036) were positively associated with 28‐day mortality in sepsis patients (Table 3). Furthermore, multivariate logistic regression further displayed that JKAP was not an independent predicting factor for 28‐day mortality in sepsis patients (OR = 0.324, *P* = 0.129), while SOFA score (OR = 5.252, *P* = 0.028) and IL‐1β (OR = 9.919, *P* = 0.023) were independent predictors of increased 28‐day mortality in sepsis patients. These data suggested that the JKAP level might contribute to decreased 28‐day mortality by influencing some clinical features such as inflammatory cytokines and disease severity.

**Table 3:** Univariate and multivariate logistic regression model analyses of factors predicting 28‐day mortality

Items	Univariate logistic regression		Multivariate logistic regression	
	*P* value	OR (95% CI)	*P* value	OR (95% CI)
JKAP level (>12.89 vs ≤12.89 pg/mL)	0.002	0.232 (0.094‐0.576)	0.129	0.324 (0.076‐1.386)
Age (>60 vs ≤60 y)	0.619	0.797 (0.326‐1.948)	0.621	1.437 (0.341‐6.057)
Gender (male vs female)	0.953	1.028 (0.416‐2.536)	0.932	1.061 (0.276‐4.078)
BMI (>24.0 vs ≤24.0 kg/m^2^)	0.619	0.797 (0.326‐1.948)	0.974	1.025 (0.235‐4.476)
Smoke (yes vs no)	0.579	1.289 (0.526‐3.162)	0.175	2.599 (0.654‐10.332)
COPD (yes vs no)	0.206	0.423 (0.112‐1.603)	0.228	0.273 (0.033‐2.249)
Cardiomyopathy (yes vs no)	0.280	0.606 (0.244‐1.503)	0.534	0.634 (0.150‐2.673)
Chronic kidney failure (yes vs no)	0.435	1.738 (0.434‐6.952)	0.293	3.078 (0.379‐25.003)
Cirrhosis (yes vs no)	0.668	1.256 (0.443‐3.561)	0.266	2.743 (0.463‐16.241)
APACHE II score (>14 vs ≤ 14)	0.036	2.533 (1.064‐6.033)	0.591	0.678 (0.164‐2.797)
SOFA score (>6 vs ≤6)	<0.001	7.407 (2.904‐18.898)	0.028	5.252 (1.193‐23.129)
Scr (>1.68 vs ≤1.68 mg/dL)	0.026	2.657 (1.125‐6.278)	0.083	4.578 (0.822‐25.492)
Albumin (>25.25 vs ≤25.25 g/L)	0.778	1.127 (0.490‐2.589)	0.047	7.163 (1.028‐49.916)
WBC (>17.20 vs ≤17.20 × 10^9^/L)	0.050	2.353 (0.999‐5.544)	0.061	5.578 (0.926‐33.592)
CRP (>105.33 vs ≤105.33 mg/L)	0.008	3.286 (1.355‐7.967)	0.495	1.687 (0.376‐7.576)
PCT (>15.10 vs ≤15.10 ng/mL)	0.018	2.857 (1.197‐6.820)	0.253	0.353 (0.059‐2.105)
TNF‐α (>213.35 vs ≤213.35 pg/mL)	0.004	3.810 (1.540‐9.423)	0.344	0.438 (0.079‐2.420)
IL‐1β (>13.59 vs ≤13.59 pg/mL)	<0.001	6.810 (2.577‐17.991)	0.023	9.919 (1.365‐72.088)
IL‐6 (>62.63 vs ≤62.63 pg/mL)	0.481	1.350 (0.586‐3.110)	0.411	0.508 (0.101‐2.554)
IL‐17 (150.04 vs ≤150.04 pg/mL)	0.036	2.500 (1.060‐5.869)	0.565	1.654 (0.298‐9.168)

Note

*P* value < 0.05 was considered significant.

Abbreviations: APACHE, acute physiology and chronic health evaluation; BMI, body mass index; CI, confidence interval; COPD, chronic obstructive pulmonary disease; CRP, C‐reactive protein; IL, interleukin; JKAP, JNK pathway–associated phosphatase; OR, odds ratio; PCT, procalcitonin; Scr, serum creatinine; SOFA, sequential organ failure assessment; TNF, tumor necrosis factor; WBC, white blood cell.

### 3.6 Correlation of JKAP expression with accumulating survival

**Figure 3:**
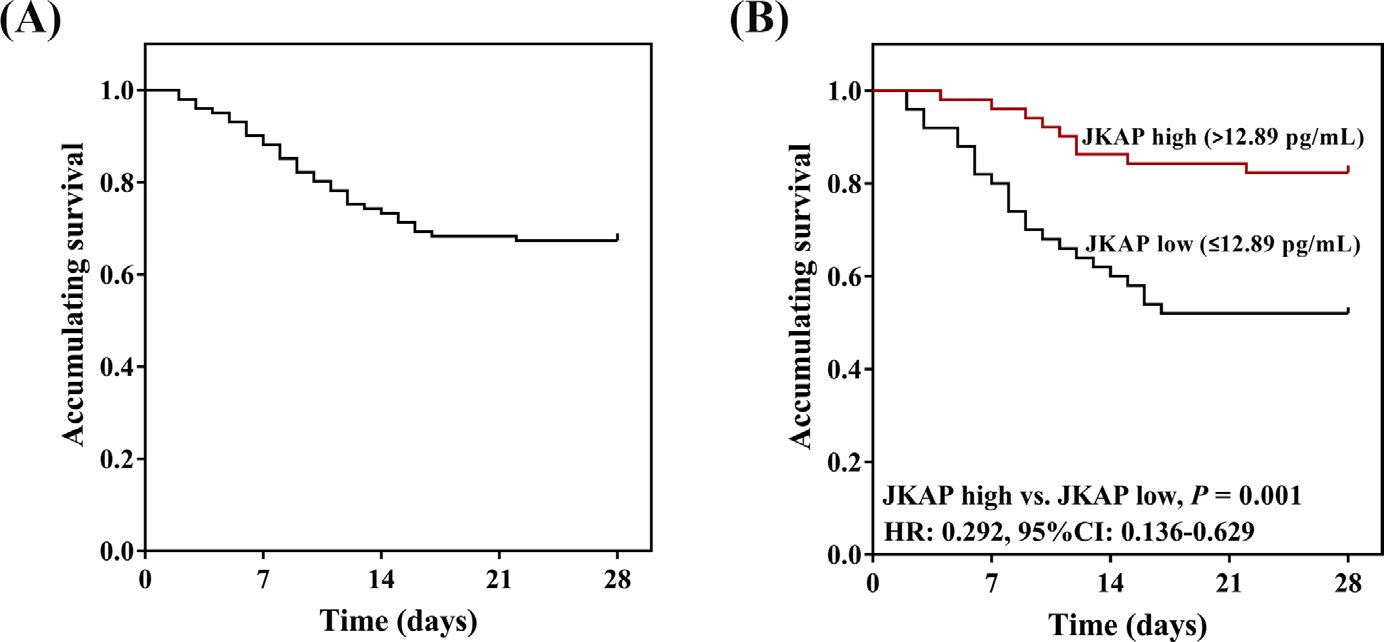
The accumulating survival of total sepsis patients, JKAP high group, and JKAP low group. The mean value of accumulating survival for total sepsis patients was exhibited by Kaplan‐Meier curve (A). And the mean value of accumulating survival was increased in JKAP high group compared with JKAP low group (B). The comparison of survival between JKAP high group and JKAP low group was conducted by log‐rank test. *P* < 0.05 was considered significant. JKAP, JNK pathway–associated phosphatase

Kaplan‐Meier curve was further drawn for assessing the accumulating survival in sepsis patients, which disclosed that the mean value of accumulating survival in total sepsis patients was 22 (95% CI: 20.2‐23.7) days (Figure 3A). Sepsis patients were then divided into JKAP high group and JKAP low group according to the median value at baseline. The accumulating survival was increased in JKAP high group (mean: 25.1 [95% CI: 23.2‐26.9] days) compared with JKAP low group (mean: 18.8 [95% CI: 16.0‐21.6] days) (*P* = 0.001; Figure 3B). These data implied that JKAP high expression predicted better accumulating survival in sepsis patients.

### 3.7 Factors affecting accumulating survival by Cox's proportional hazards regression model analyses

Univariate Cox's proportional hazards regression presented that JKAP level (HR = 0.292, *P* = 0.002) was positively associated with accumulating survival in sepsis patients (Table 4). However, APACHE II score (HR = 2.208, *P* = 0.032), SOFA score (HR = 5.095, *P* < 0.001), Scr (HR = 2.184, *P* = 0.031), CRP (HR = 2.369, *P* = 0.020), PCT (HR = 2.447, *P* = 0.015), TNF‐α (HR = 2.316, *P* = 0.023), and IL‐1β (HR = 4.920, *P* < 0.001) were negatively associated with accumulating survival in sepsis patients. And multivariate Cox's regression was further conducted, which displayed that JKAP level was not an independent risk factor for better accumulating survival in sepsis patients (HR = 0.660, *P* = 0.414), while SOFA score (HR = 4.297, *P* = 0.011) and IL‐1β (5.820, *P* = 0.015) were independent predicting factors for worse accumulating survival in sepsis patients. These data indicated that JKAP might lead to increased accumulating survival by affecting some clinical features.

**Table 4:** Univariate and multivariate Cox's proportional hazards regression model analyses of factors affecting accumulating survival during the study

Items	Univariate Cox's regression		Multivariate Cox's regression	
	*P* value	HR (95% CI)	*P* value	HR (95% CI)
JKAP level (>12.89 vs ≤12.89 pg/mL)	0.002	0.292 (0.136‐0.629)	0.414	0.660 (0.244‐1.787)
Age (>60 vs ≤60 y)	0.575	0.809 (0.385‐1.699)	0.231	0.550 (0.207‐1.462)
Gender (male vs female)	0.985	1.007 (0.479‐2.117)	0.824	1.111 (0.440‐2.806)
BMI (>24.0 vs ≤24.0 kg/m^2^)	0.732	0.879 (0.418‐1.846)	0.865	0.912 (0.316‐2.632)
Smoke (yes vs no)	0.612	1.206 (0.585‐2.488)	0.828	1.107 (0.442‐2.774)
COPD (yes vs no)	0.257	0.504 (0.154‐1.650)	0.630	0.693 (0.156‐3.082)
Cardiomyopathy (yes vs no)	0.289	0.661 (0.307‐1.421)	0.576	0.764 (0.297‐1.964)
Chronic kidney failure (yes vs no)	0.261	1.822 (0.640‐5.188)	0.104	3.799 (0.761‐18.967)
Cirrhosis (yes vs no)	0.798	1.115 (0.484‐2.570)	0.840	1.122 (0.369‐3.405)
APACHE II score (>14 vs ≤14)	0.032	2.208 (1.070‐4.555)	0.565	0.741 (0.267‐2.059)
SOFA score (>6 vs ≤ 6)	<0.001	5.095 (2.361‐10.994)	0.011	4.297 (1.406‐13.128)
Scr (>1.68 vs ≤1.68 mg/dL)	0.031	2.184 (1.074‐4.443)	0.073	2.700 (0.910‐8.009)
Albumin (>25.25 vs ≤25.25 g/L)	0.756	1.114 (0.563‐2.206)	0.215	2.021 (0.664‐6.149)
WBC (>17.20 vs ≤17.20 × 10^9^/L)	0.081	1.880 (0.925‐3.824)	0.972	1.019 (0.355‐2.921)
CRP (>105.33 vs ≤105.33 mg/L)	0.020	2.369 (1.148‐4.889)	0.444	1.533 (0.513‐4.578)
PCT (>15.10 vs ≤15.10 ng/mL)	0.015	2.447 (1.186‐5.050)	0.292	0.500 (0.138‐1.814)
TNF‐α (>213.35 vs ≤213.35 pg/mL)	0.023	2.316 (1.123‐4.778)	0.433	0.642 (0.212‐1.946)
IL‐1β (>13.59 vs ≤ 13.59 pg/mL)	<0.001	4.920 (2.132‐11.355)	0.015	5.820 (1.404‐24.131)
IL‐6 (>62.63 vs ≤62.63 pg/mL)	0.424	1.323 (0.667‐2.625)	0.225	0.513 (0.174‐1.507)
IL‐17 (150.04 vs ≤150.04 pg/mL)	0.019	2.372 (1.150‐4.895)	0.293	1.941 (0.564‐6.674)

Note

*P* value < 0.05 was considered significant.

Abbreviations: APACHE, acute physiology and chronic health evaluation; BMI, body mass index; CI, confidence interval; COPD, chronic obstructive pulmonary disease; CRP, C‐reactive protein; HR, hazard ratio; IL, interleukin; JKAP, JNK pathway–associated phosphatase; PCT, procalcitonin; Scr, serum creatinine; SOFA, sequential organ failure assessment; TNF, tumor necrosis factor; WBC, white blood cell.

## 4 DISCUSSION

In the present study, we observed that (a) JKAP level was negatively correlated with sepsis risk, and decreased JKAP level was associated with higher disease severity and inflammation in sepsis patients. (b) JKAP had good value in distinguishing deaths from survivors, and its high expression was associated with decreased 28‐day mortality and more prolonged accumulating survival, while SOFA score and IL‐1β independently predicted increased 28‐day mortality and worse accumulating survival in sepsis patients.

Sepsis is a complex disease united by the stimulation of systemic inflammation and the host response to infection.^13^ Timely recognition is the crucial issue of sepsis, which is based on non‐specific physiological criteria and identification of the infection, whose failure often causes the delay in the diagnosis and generating treatment, leading to unfavorable outcomes in sepsis patients.^5^ Additionally, the sepsis patients who are admitted in ICU often face unacceptably high short‐term mortality and undesirable prognosis in the long term due to advanced disease severity and accompanying disease complications, which leads to a considerable consumption of healthcare resources and substantial burden for society.^14^ Thus, it is essential to explore potential new biomarkers as adjuvants for recognizing sepsis risk and identifying the sepsis patients at high risk of having a poor prognosis.

JKAP is a member of DUSPs family, which is a group of cysteine‐based protein tyrosine phosphatases, and is involved in the pathogenesis of several human diseases.^15^ There has been evidence in the last decade that JKAP regulates autoimmunity in various diseases through inactivating T‐cell receptor signaling and further suppressing T‐cell immune responses.^6^, ^15^ For example, a study reveals that JKAP in peripheral blood T cells is downregulated in SLE patients compared to health controls; besides, its downregulation is correlated with increased SLE disease activity index in SLE patients and is associated with higher daily urinary protein amounts and poorer renal outcomes in SLE‐nephritis patients.^9^ In another study, JKAP in mucosa is decreased in active IBD patients compared to IBD patients at remission and health controls and is negatively correlated with disease activity as well as systemic inflammation in IBD patients; also, JKAP is observed to suppress CD4^+^ T‐cell activation and Th1/Th17 Cell differentiation in IBD.^8^ Given that SLE and IBD both belong to immune and inflammatory diseases, we speculated that the expression of JKAP might also be dysregulated and play a critical role in sepsis. We found that JKAP had the potential for predicting decreased sepsis risk and downregulation of JKAP was associated with exacerbated sepsis and higher level of inflammatory cytokines, indicating that JKAP might be a candidate biomarker for disease risk and severity of sepsis. These might be explained by the following reasons: (a) Downregulation of JKAP enhanced T cell–mediated immunity and autoimmunity by activating T‐cell receptor signaling, which accelerated the development and progression of sepsis. (b) Downregulation of JKAP might promote the release of inflammatory cytokines via inactivating JNK signaling pathways, leading to activation of inflammatory cytokine–induced immune responses and advanced disease severity in sepsis patients. However, the underlying mechanism of JKAP in regulating immune and inflammatory responses in sepsis needs further validation.

Currently, there is limited study focusing on the association of JKAP and prognosis in diseases related to immunity and inflammation. Only one paper indicates that high expression of JKAP predicts a better response to anti‐TNF‐α treatment in IBD patients.^8^ In our study, we found that JKAP expression was associated with disease risk and severity of sepsis; thus, we speculated that JKAP might be of prognostic importance in sepsis patients. Therefore, we conducted further assessments and discovered that JKAP level was negatively correlated with survival, while it was not able to independently predict poor survival in sepsis patients. These could be explained by the following: (a) Downregulation of JKAP might promote T cell–mediated immunity and autoimmunity by upregulating T‐cell receptor signaling, which enhanced the activation of inflammatory responses and increased disease severity, resulting in poor survival in sepsis patients. (b) Decreased JKAP level might interact with other clinical features, such as inflammatory cytokines and advanced disease severity, to influence the survival; hence, JKAP was not an independent predictive factor for survival in sepsis patients.^6^ This might be supported by one of our findings which elucidated that JKAP was negatively correlated with APACHE II score, SOFA score, TNF‐α, IL‐1β, IL‐6, and IL‐17, and that SOFA score and IL‐1β were independent predictive factors for decreased survival in sepsis patients. SOFA score reflects the facets of organ dysfunction; therefore, the sepsis patients with high SOFA score might have more severe organ dysfunction, which contributed to the undesirable survival.^16^, ^17^ Additionally, IL‐1β was a well‐known proinflammatory cytokine; therefore, the high expression of IL‐1β might lead to the activation of the inflammasome response, as well as predict the worse survival in sepsis patients.^18^

There were several limitations to our studies. (a) The sample size of this study was relatively small, which might influence the statistic power. (b) Accumulating survival was calculated from the day of admission to the day of death in the hospital or last visit, while the long‐term effect of JKAP level on survival needed to be observed in a more extended follow‐up period. (c) The underlying mechanism of JKAP in the sepsis was not explored in our study; thus, further experiments were needed.

In summary, JKAP is of good value in predicting lower sepsis risk and its downregulation correlates with advanced disease severity, higher level of systemic inflammation, and poor survival in sepsis patients.
